# Retinoic acid receptor antagonists for male contraception: current status^†^

**DOI:** 10.1093/biolre/ioaa122

**Published:** 2020-07-15

**Authors:** Md Abdullah Al Noman, Jillian L Kyzer, Sanny S W Chung, Debra J Wolgemuth, Gunda I Georg

**Affiliations:** 1 Department of Medicinal Chemistry, College of Pharmacy, Institute for Therapeutics Discovery and Development, University of Minnesota, Minneapolis, MN, USA; 2 Department of Genetics and Development, Columbia University Irving Medical Center, New York, NY, USA; 3 Department of Obstetrics and Gynecology, Columbia University Irving Medical Center, New York, NY, USA; 4 The Institute of Human Nutrition, The Herbert Irving Comprehensive Cancer Center, Columbia University Irving Medical Center, New York, NY, USA

**Keywords:** retinoic acid receptor, antagonists, selectivity, male contraception, mouse model, structure–activity relationships

## Abstract

Retinoic acid receptor alpha (RARA), a nuclear receptor protein, has been validated as a target for male contraception by gene knockout studies and also pharmacologically using a pan-retinoic acid receptor antagonist. Retinoic acid receptor alpha activity is indispensable for the spermatogenic process, and therefore its antagonists have potential as male contraceptive agents. This review discusses the effects of systematic dosing regimen modifications of the orally bioavailable and reversible pan-antagonist **BMS-189453** as well as studies with the alpha-selective antagonists **BMS-189532** and **BMS-189614** in a murine model. We also provide an overview of structure–activity studies of retinoic acid receptor alpha antagonists that provide insight for the design of novel alpha-selective ligands.

## Teaser

Fertility studies with retinoic acid receptor antagonists in male mice and the current state of RARA-selective antagonist development are reviewed.

## Introduction

Despite making progress worldwide in providing birth control options to families, the rate of unintended pregnancies, defined as both unwanted and mistimed pregnancies resulting from not using contraceptives or incorrect/inconsistent contraceptive use, remains high [1]. While rates of accidental pregnancies have decreased, the unintended pregnancy rates in developed nations are 45% and remain around 65% in developing nations [1]. Approximately 56% of all unintended pregnancies ended in abortion between 2010 and 2014—55% in developing nations and 59% in developed nations [1]. There is thus a critical need for additional approaches and resources for reversible contraception. While many reversible contraceptive methods are available to women, such as hormonal birth control, emergency contraception, vaginal rings, cervical caps, and spermicides, reversible methods for men are limited to condoms and withdrawal. For in-depth reviews and discussions of male contraceptives, refer to Long et al. [2] and Blithe [3]. There has been interest in the use of testosterone and various testosterone esters as potential contraceptive agents [4, 5]; however, testosterone alone does not completely suppress sperm production, and there are ethnic differences in its efficacy [4, 6]. Supplementation of testosterone administration with progestogens enhances the suppression of sperm production at lower doses of testosterone. However, the effects of long-term exogenous testosterone administration remain unclear. Treatment with testosterone has been associated with several negative side effects, which can include cardiac toxicity [7] and liver damage [8]. The most common negative side effect was erythrocytosis, which has been linked to cerebrovascular disease [9]. Additionally, exogenous testosterone has been shown to lower high-density lipoprotein (HDL) cholesterol and increase hematocrit, hemoglobin, and thromboxane, all of which are associated with cardiovascular disease [7]. In addition to the more serious side effects, patients also experienced weight gain, acne, injection-site pain, and mood changes like aggression and decreased libido [10]. In the study described above, 2.2% of patients failed to reach the oligozoospermia threshold, indicating that certain men are “nonresponders” to testosterone treatment [10].

**Figure 1:**
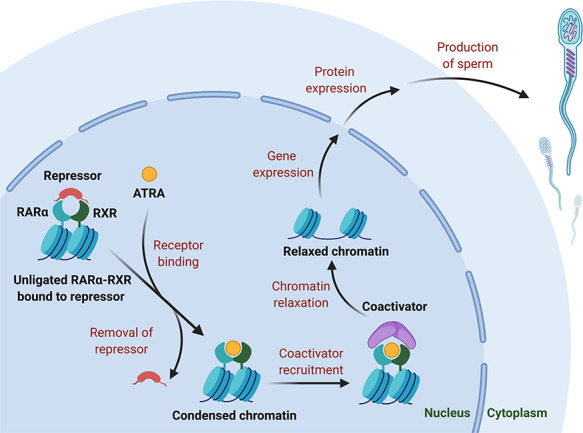
Molecular mechanisms underlying the role of RARα in regulating gene expression during spermatogenesis. The RAR alpha–RXR complex with repressor proteins inhibits gene transcription by facilitating chromatin condensation. After binding with ATRA, repressor proteins dissociate from the complex and coactivators are recruited, resulting in chromatin relaxation and expression of genes necessary for male germ cell differentiation during mammalian spermatogenesis.

Therefore, a critical need exists for an effective, nonsteroidal hormone-based reversible male contraceptive that exhibits few if any side effects, health risks, and further complications. While hormone therapy relies on interrupting the spermatogenic process, there are far more targets for non-hormonal-based therapies that can be pursued [11]. Non-hormonal male contraceptive approaches involve targeting proteins that affect either sperm production or sperm function and are anticipated to have minimal side effects, depending on the specificity and potency of inhibitors for the target protein. This review will not attempt to provide a comprehensive overview of hormonal and non-hormonal male contraception but will focus on one attractive target, the retinoic acid receptor alpha (RARA), as its ligand all-*trans*-trans retinoic acid (ATRA) is required for several phases of spermatogenesis, including differentiation of type A spermatogonia, the onset of meiosis, and spermiation [12, 13]. This review will discuss RARA, in particular, as a target and will explore what has been learned from systematic dosing regimens of antagonists to the receptor in a murine model. Furthermore, the review will provide an overview of structure–activity studies of existing RARA inhibitors, which are instructive for the design of novel potent and selective retinoid antagonists for reversible male contraception.

**Figure 2:**
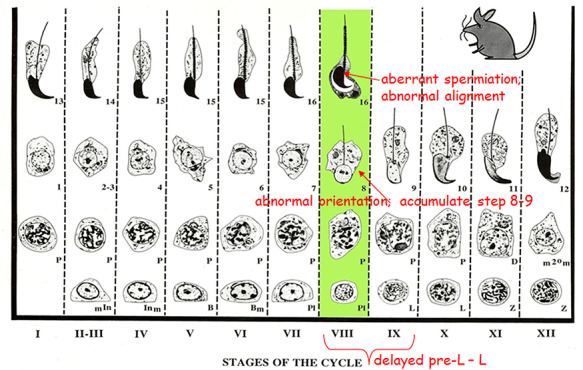
Cartoon representation of the mouse spermatogenic cycle illustrating the profound abnormalities in RAR alpha-deficient testes clustered in stage VIII–IX tubules. Details of the symbols used in the staging diagram can be found in Russell et al. [62]. The green bar line indicates stage VIII, which showed the highest frequency of cellular abnormalities. Red arrows point to the specific cell types at stage VIII, in which various abnormalities were found. Reprinted with permission from the article by Wolgemuth and Chung [21].

## Implications of retinoid signaling for male contraception

Previous studies have demonstrated that retinoid signaling is required for the differentiation of undifferentiated type A_aligned_ spermatogonia to differentiating type A_1_ spermatogonia [12, 14, 15] and recently for aspects of meiosis in spermatocytes [16, 17]. In addition, spermiation is exquisitely sensitive to perturbation of retinoid signaling [18–22].

ATRA, the active metabolite, is formed via the metabolic oxidation of dietary retinol (vitamin A). All-*trans*-retinol is first oxidized to all-*trans*-retinaldehyde by alcohol dehydrogenase or retinol dehydrogenase and then subsequently oxidized by aldehyde dehydrogenase to ATRA [14]. RA exerts its effects through binding to a family of nuclear receptors comprising the RARs as well as the retinoid X receptors (RXRs) (Figure 1) [20]. These receptors have three isoforms: alpha, beta, and gamma [20]. RARs are primarily activated through the binding of ATRA, while RXRs are exclusively activated by 9-*cis* retinoic acid [20]. These receptors are active as dimers, either as RXR/RXR homodimers or RXR/RAR heterodimers [20]. The dimers bind to RA response elements in the DNA to affect transcription [20]. In the absence of RA, histone deacetylases and other repressors of transcription are recruited, keeping DNA tightly bound to the histone (heterochromatin) to prevent transcription [14]. Once activated by RA, the complex recruits histone acetyltransferases and other coactivators that acetylate lysines and thereby neutralize the interaction between the negatively charged DNA and the positively charged histone; the tightly packed heterochromatin then unfolds to form transcription-ready lightly packed euchromatin, resulting in gene transcription (Figure 1) [14, 23].

The need for vitamin A and its active metabolite ATRA for normal spermatogenesis has been recognized for decades [24–26]. There are numerous excellent and comprehensive reviews on the role of vitamin A, its synthesis, transport, metabolism, and downstream function, and the potential interference with its function as approaches to male contraception to which the reader is referred [2–5, 12, 14, 17, 20, 21]. As mentioned above, metabolism of dietary vitamin A involves the conversion of retinol to retinal and finally to ATRA. Inhibitors of enzymes involved in this process could potentially disrupt ATRA synthesis and thereby stop spermatogenesis. Indeed, suppression of spermatogenesis involving the ATRA pathway was demonstrated with a bisdichloroacetyldiamine analog (BDAD) [2, 27, 28]. WIN 18 446, one of the BDAD analogs, was used in a clinical study to treat over 60 men for 1 year and was shown to efficiently suppress sperm production in men [27]. However, men taking WIN 18 446 exhibited a severe negative disulfiram reaction upon alcohol consumption, resulting in termination of development of the drug. The mechanism of suppression was subsequently elucidated when WIN 18 446 was shown to inhibit the enzymes aldehyde dehydrogenase ALDH1A1 and ALDH1A2 [29, 30]. Using X-ray crystallography, direct binding studies, and enzymatic analyses, a recent report determined the structural basis of ALDH1A2 inhibition by WIN 18 446, and two novel and reversible small molecule inhibitors were identified which are being investigated for their property of not blocking alcohol metabolism [28, 31]. Results provide a structural framework toward the rational design of potent and selective ALDH1A2 inhibitors that might be suitable for non-hormonal male contraception [28].

**Table 1:** Structures and antagonist data for RAR inhibitors **BMS-189453** (**1**) [35], **BMS-189532** (**2**) [37], and **BMS-195614** (**3**) [38].

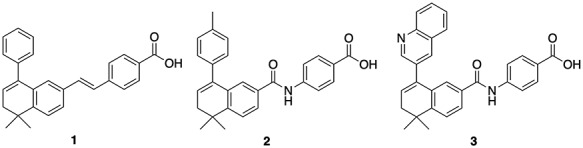						
	Antagonism IC_50_ (nM)^a^			*K*_d_ (nM)		
Compound	RARA	RARB	RARG	RARA	RARB	RARG
**1 (BMS-189453)**	200	200	200	—	—	—
**2 (BMS-189532)**	—	—	—	27	1020	3121
**3 (BMS-195614)**	500	5000	10 000	—	—	—

^a^Transactivation assay.

**Figure 3:**
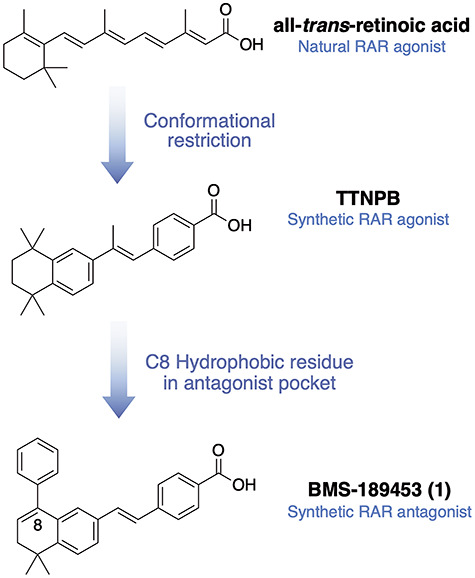
Development of RAR antagonists from all-*trans-*retinoic acid.

The present review is focused on the unique functions of the RARs and RARA in particular. In this light, we note the important insight into the molecular basis for the need of this micronutrient that was revealed by targeted mutagenesis of *Rara*, the gene encoding RARA. In *Rara−/−* mice, there was a disruption in spermatogenesis with defects similar to the effects of VAD and complete male infertility while the females were fertile [20, 32]. The majority of testicular tubules at 4–5 months of age were severely abnormal, with few germ cells, and the epididymides contained only very few abnormal spermatids [32]. A study determining the chronology of appearance of spermatogenic abnormalities in *Rara−/−* testes showed that in the tubules that did contain germ cells, spermiation was defective [18]. Late spermatids are normally released at the end of stage VIII of the spermatogenic process; however, late spermatids in these mice were still present at the luminal edge at stage IX (Figure 2) [18, 19]. Spermatids were also observed undergoing apoptosis and engulfment by the Sertoli cells [19]. A study of germ-cell-specific *Rara* conditional knockout mice (*Rara* cKO) demonstrated that RARA is involved in modulating the synaptonemal complex of proteins that forms between homologous chromosomes during meiosis, which is consistent with the previous observation that meiosis is delayed in VAD animals and *Rara−/−* mice [16]. The role of RARA during spermiogenesis in the germline is further supported by the partial rescue of spermiogenic defects by targeted expression of *Rara* cDNA specifically in haploid spermatids of otherwise RARA-deficient mice [22]. Another recent study has also shown that ATRA plays a role at two postmeiotic transitions: the initiation of spermatid elongation and spermiation [17, 33]. For a more detailed review of the functions that are important specifically during spermatogenesis and the basic cellular processes within testicular cells that may be regulated by retinol and ATRA, with a focus on cells within the seminiferous tubules, please refer to Chung and Wolgemuth [20] and Endo et al. [17].

**Figure 4:**
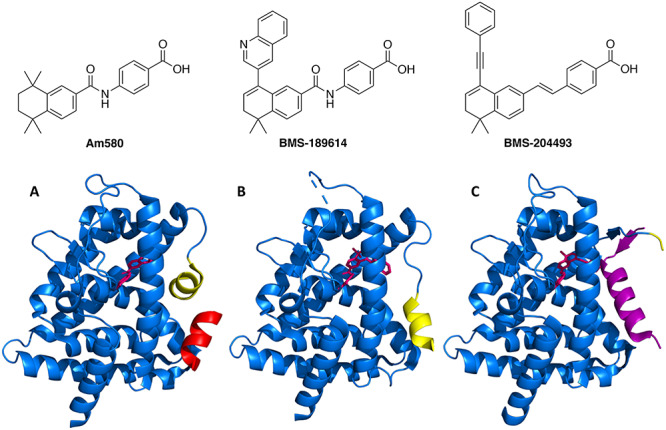
Crystal structures of RARA with different ligands. (A) Agonist-bound RAR shows H12 (yellow) closing the entrance to the LBD and creating a binding site for the coactivator (red) (PDB ID: 3kmr). (B) Antagonist binding prevents H12 (yellow) binding to the LDB entrance due to the sterically demanding substituent on the ligand molecule so that the coactivator cannot bind (PDB ID: 1dkf). (C) Binding of an inverse agonist prevents H12 (yellow) binding to the LDB entrance and results in a highly mobile structure of H12; also it facilitates the binding of a corepressor fragment (magenta) (PDB ID: 3kmz).

**Figure 5:**
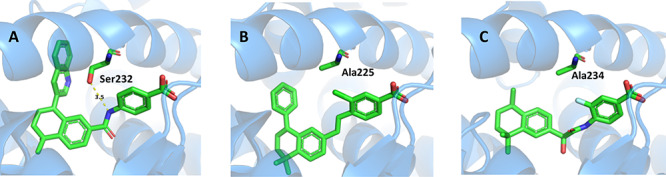
Structural differences in the ligand-binding domain of RARA ((A) PDB ID, 1dkf), RARB ((B) PDB ID, 4jyi), and RARG ((C) PDB ID, 4lbd). Ser232 is unique in RARA, whereas the same position is occupied by alanine residues 225 and 234 in RARB and RARG, respectively.

## Oral administration of pan-RAR antagonist **BMS-189453** reversibly inhibits spermatogenesis in mice

Studies showing “testicular toxicity” but no other side effects of several retinoid antagonists [34] suggested that RAR antagonists might be attractive compounds for a non-hormonal approach to male contraception. Such a pharmacological approach to inhibit spermatogenesis in the mouse model was explored using low doses (initially 5 mg/kg for 7 days) of the pan-RAR antagonist **BMS-189453** (**1**, Table 1) [35]. Morphological examination of testes from the treated animals showed that spermatogenesis was disrupted, with a failure of spermatid alignment and sperm release as well as loss of germ cells into the lumen, abnormalities that resembled those in VAD and *Rara−/−* testes. Most importantly, the induced sterility was reversible as assessed by mating studies. There were no changes in testosterone levels, suggesting that the drug would not affect male libido or sexuality. Enhanced efficacy and a lengthened infertility period with full recovery of spermatogenesis were observed using systematically modified dosing regimens (5 mg/kg for 2 weeks versus 2.5 mg/kg for 4 weeks). Toxicological evaluation including hematology, serum chemistry, and hormonal and pathological evaluations was performed blinded in collaboration with Dr. Stephen Griffey, from the Comparative Pathology Laboratory, University of California Davis, and revealed no detectable abnormalities or adverse side effects except the distinct testicular pathology [35]. Finally, the resulting progeny of two recovered males (4 litters, 22 males and 22 females) were allowed to grow to adulthood. These progeny were healthy, and the males exhibited normal testicular weight, spermatogenesis, and serum testosterone levels [35]. Both male and female progeny yielded a similar number of embryos upon mating. Because tailoring the dose-to-weight on a daily basis would not be practical for application in humans, a regimen in which the dose was modified weekly was examined. Induction of sterility and recovery of fertility were observed and were identical to those obtained with a regimen modified daily. These collective results suggest that testes are exquisitely sensitive to disruption of retinoid signaling and that RAR antagonists may represent new lead molecules in developing reversible nonsteroidal male contraceptives.

**Table 2:** Structure and data for RARA-selective antagonist **4** [53].

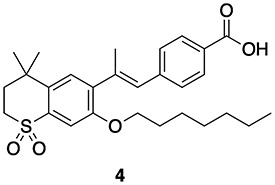			
	Binding IC_50_ (nM)		
Compound	RARA	RARB	RARG
ATRA	14	14	14
**4 (Ro 41-5253)**	60	2400	3300

## Prolonged oral administration of a pan-RAR antagonist inhibits spermatogenesis with a rapid recovery

It was encouraging that oral administration of the pan-retinoic acid receptor antagonist **1** reversibly inhibited spermatogenesis, with no detectable side effects. Given that contraception is typically used for long periods of time, the next experiments focused on determining the lowest dose and the longest dosing period regimens that inhibit spermatogenesis but also result in complete restoration of fertility upon cessation of administration of the drug [36]. The studies demonstrated that daily doses of **1** as low as 1.0 mg/kg with dosing periods of 4, 8, and 16 weeks resulted in 100% sterility in all regimens, with restoration of fertility upon cessation of the drug treatment even for as long as 16 weeks. There was no change in testosterone levels in these males. The progeny examined from two of the recovered males were healthy and fertile, with normal testicular weight and testicular histology.

Strikingly, a more rapid recovery, as assessed by mating studies, was observed at the lower dose and longer dosing periods (1 mg/kg for 16 weeks) [36]. Insight into possible mechanisms underlying this rapid recovery was obtained at two levels. First, histological examination revealed that spermatogenesis was not as severely disrupted at the lower dose and with the longer treatment regimens. Second, gene expression analysis revealed that the more rapid recovery may involve the interplay of ATP-binding cassette efflux and solute carrier influx transporters in the testes. These observations showed that (i) increasing the duration of treatment still resulted in restoration of fertility following cessation of treatment; (ii) even lower doses of the drug can be effective in inhibiting spermatogenesis; and (iii) longer dosing periods were characterized by a more rapid restoration of spermatogenesis and fertility [36].

A next logical question is whether such pan-RAR antagonists (or, if available, RARA-selective antagonists) will function in higher mammals by testing the drug in a nonhuman primate model and assessing the efficacy of the antagonists in the induction of sterility and restoration of fertility. This research may have a potentially important impact by identifying a prospective therapeutic for male contraception.

## RARA-selective antagonists may allow a better therapeutic efficacy in reversibly inhibiting spermatogenesis

After a successful demonstration of contraceptive effect by pan-RAR antagonist **1** (**BMS-189453**), RARA-selective antagonists were pursued to preclude effects on RARB and RARG functions. Two RARA-selective antagonists, **BMS-189532** (**2**) [37] and **BMS-189614** (**3**) [38] (Table 1), were characterized by transactivation and transactivation competition assays and were tested in a mouse model to determine whether they effectively inhibit spermatogenesis. These studies showed that although these two antagonists were potent in vitro, they displayed poor in vivo activity in mice when administered orally at the dose of 2 mg/kg and 10 mg/kg for 7 days [38]. Testicular weights were normal, and morphological analysis revealed normal spermatid alignment and sperm release. To assess properties that might have resulted in the poor in vivo efficacy of these compounds, in vitro drug property analyses were performed with one of these antagonists, compound **2**, and compared with the pan-antagonist **1**. The discrepancies may be explained by several factors, including high plasma protein binding, faster hepatic metabolism relative to the pan-antagonist, and only moderate permeability [38]. The conclusion of poor oral bioavailability was supported by more pronounced defects in mice when the antagonist was administered intravenously versus intraperitoneally. These results are crucial for designing new RARA-selective antagonists for pharmaceutical application.

**Table 3:** Structures and data for chromene and tetrahydronaphthalene RAR antagonists [37].

					*K*_i_ (nM)		
Compound	X	R	Y	Z	RARA	RARB	RARG
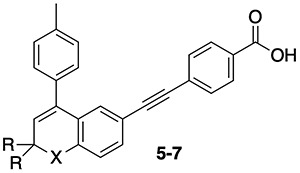							
ATRA					15	13	18
**5 (AGN 193109)**	CMe_2_	H	—	—	16	7	7
**6**	O	CH_3_	—	—	14	5	14
**7**	S	CH_3_	—	—	4	2	10
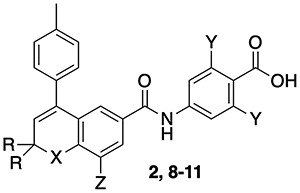							
**2 (AGN 193491)**	CMe_2_	H	H, H	H	27	1020	3121
**8 (AGN 193618)**	CMe_2_	H	H, F	H	5.7	622	863
**9 (AGN 194202)**	CMe_2_	H	F, F	H	32	2256	>30 000
**10 (AGN 194301)**	O	CH_3_	H, F	Br	2.8	320	7258
**11 (AGN 194574)**	O	CH_3_	F, F	Br	1.5	898	10 618

**Table 4:** Structures of pyrazole agonist **12**, pyrazole agonist **13** (**ER-27191),** pyrrole antagonist **14** [57], and structure–activity relationship data for quinolone pyrrole antagonists **15**–**17** [58].

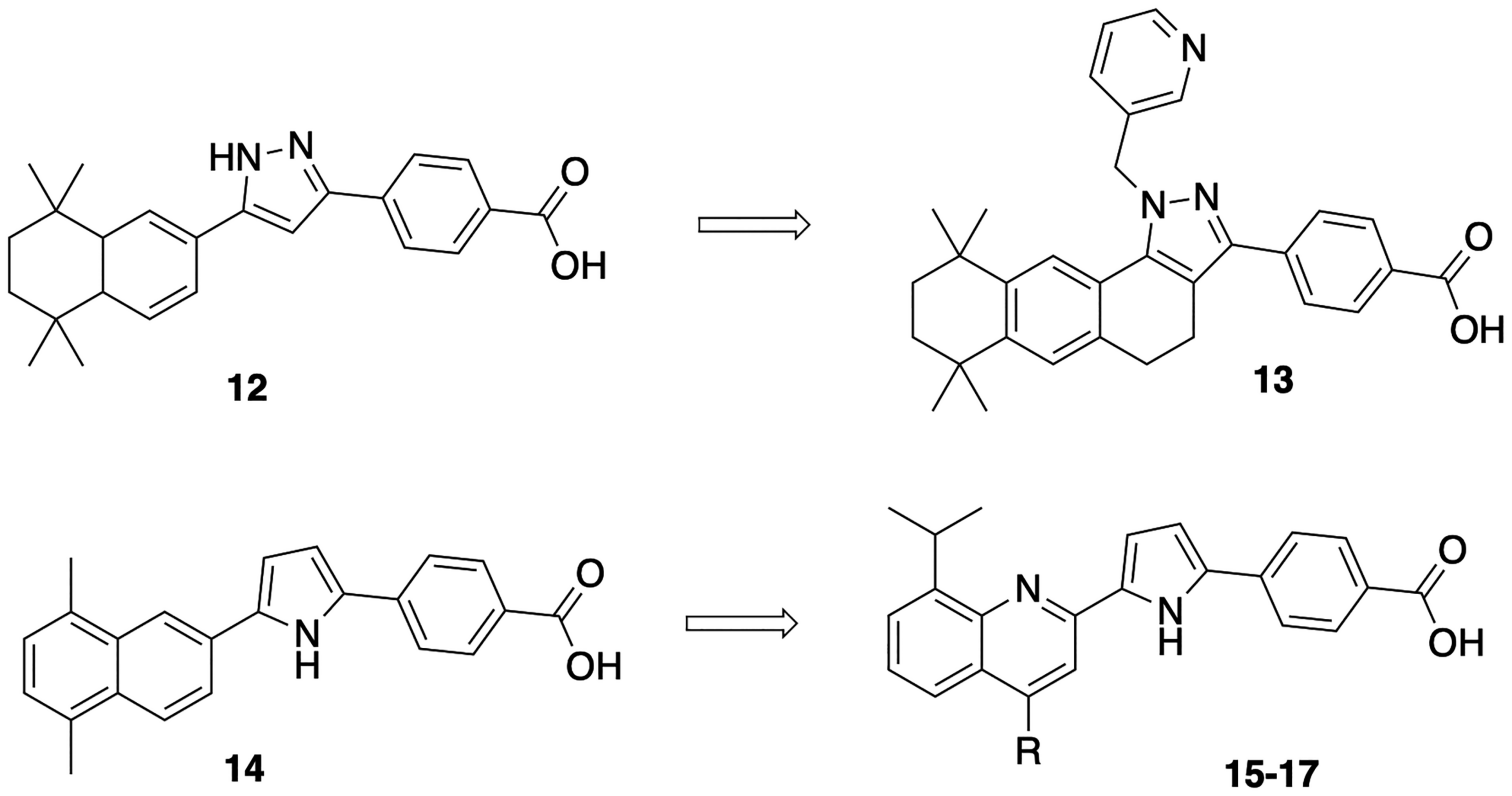					
		Binding affinity IC_50_ (nM)			Antagonism IC_50_ (nM)
Compound	R	RARA	RARB	RARG	
**15**	MeO	0.5	247	350	1.6
**16**	Et	0.5	17	9	17
**17 (ER 50891)**	Ph	1.2	278	181	6.4

## The medicinal chemistry of RAR inhibitors

In the following section, we will review structure–activity relationships for agonism, antagonism, and inverse agonism for the three RARs. Insights from these studies are expected to be useful for the design of novel RARA-specific antagonists with suitable pharmacokinetic properties.

The development of RAR ligands began in the 1980s to obtain potent RAR agonists with selectivity toward RAR over RXR. During initial development, conformational restriction was used to decrease rotational freedom of ATRA. The polyene chain next to the cyclohexene moiety was converted to an aromatic ring and the terminal dienoic acid to a benzoic acid moiety (Figure 3). This provided E)-4-(2-(5,5,8,8-tetramethyl-5,6,7,8-tetrahydronaphthalen-2-yl)prop-1-en-1-yl)benzoic acid, a chemically stable and potent RAR agonist that does not bind RXR [39]. The introduction of bulky substituents at the 8-position of an agonist molecule (such as the phenyl group in compound **1**) led to antagonism and inverse agonism [40–45].

In its native unliganded conformation, helix H12 of RAR adopts a conformation that prevents coactivator binding but allows corepressor binding, thereby inhibiting gene transcription [40, 42]. After agonist binding, the H12 helix conformational change prevents access to the ligand-binding domain (LBD) while generating a coactivator binding site to which coactivators are recruited that promote gene transcription (Figure 4A). As shown above for antagonist **1**, an RAR agonist can be modified to become an antagonist by introducing a sterically demanding substituent. This tactic has been successfully employed for a number of ligands [46–48]. It was later discovered that the phenyl moiety is not large enough to induce complete antagonism and results in partial antagonism, whereas a tolyl group confers full antagonism [49]. Sterically demanding substituents present on the antagonist cause helix H12 to adopt a conformation that prevents coactivator binding. The crystal structures of agonists and antagonists bound to RARA (Figure 4) provide insight into protein conformational changes depending on which ligand is bound. When antagonist Am580 is bound to RARA (Figure 4B), the H12 helix fails to cap the LBD and instead binds to the coactivator binding site resulting in coactivator recruitment failure [41, 44]. When mixed agonists bind to the LBD, H12 can assume agonist and antagonist conformations due to inadequate steric bulk that is required to completely inhibit H12 coactivator binding [45, 50]. In the case of inverse agonists, ligand binding stabilizes corepressor binding with RAR and further inhibits basal levels of gene transcription. The structural requirement for inverse agonism is less straightforward due to the highly variable nature of the H12 domain and the requirement for suitable H12 conformation for repressor binding (Figure 4C) [41].

### RARA selectivity

A common structural feature for both agonists and antagonists among RARA-selective ligands is the presence of a hydrogen bond donor such as an amide (**2**, **8**–**11**, Table 3) or a pyrrole (**15**–**17**; Table 4) interacting with Ser232 of RARA (Figure 5A) [51]. This selectivity can be explained by the structural differences between the three RARs. A comparison of their binding domains reveals the presence of Ser232 in RARA, but in the analogous position of both RARB and RARG, there is an alanine (Figure 5B and C). Therefore, the more polar binding site architecture of RARA favors the binding of polar groups. Moreover, substitution of Ser232 with alanine abolishes the selectivity of ligands toward RARA [52].

### Selective RARA antagonists

The first RARA-selective antagonist, compound **4** (**Ro 41-525**) (Table 2), was reported in 1992 [53]. The compound has little structural similarity with other antagonists because it does not carry the sterically demanding substituent characteristic of the other antagonists. Instead, the compound has a heptyl ether chain *ortho* to the olefinic linkage. Additionally, it lacks the hydrogen bond donor commonly found in other RARA-selective antagonists. This deviation from the normal architecture suggested that the compound might exploit other structural differences in the RAR proteins to achieve selectivity.

Initially, phenylacetylene analogs **5** (**AGN 193109**), **6**, and **7** were described as potent RAR antagonists that do not bind to RXRs (Table 3) [54]. Results from a transactivation assay showed that compound **5** binds to the RAR–RXR dimer and inhibits RA-induced gene expression in a dose-dependent manner. Evaluation of analogs in which the tolyl group was replaced with other substituted aromatic rings revealed that the *para*-substitution of phenyl rings with methyl, ethyl, and methoxy groups provided maximal activity [55]. Further truncation or elaboration of the *para*-substituent did not improve activity and confirmed the optimal activity for 4-methylphenyl and 4-ethylphenyl substituents on the aromatic rings [56]. Chromene and thiochromene substitution for the tetrahydronaphthalene ring also was explored. Thiochromenes were more potent than chromenes, and analogs carrying *gem*-dimethyl groups were superior to demethyl analogs. When the acetylene linker in tetrahydronaphthalene and chromene scaffolds of pan-RAR inhibitors **5** and **6** were replaced with the hydrogen bond donating amide linker, the resulting compounds **2** and **8**–**11** (Table 3) were found to selectively antagonize the activity of RARA over RARB and RARG [37]. This exemplifies how non-selective antagonists can be modified to gain RARA selectivity. It was also reported that fluoro substitution on the *ortho-*position of the benzoic acid moiety increased the alpha selectivity of the antagonists, which increased further with difluoro substitution (**8**–**9** and **10**–**11**).

A similar approach to obtain an antagonist from a reported agonist was adopted to identify RARA-selective antagonist **15** (Table 4). Conformational restriction of agonist **12** (500 nM antagonism) [47] by introducing another ring and by adding a sterically demanding 3-pyridin-3-ylmethyl group to the N1-pyrazole moiety provided antagonist **13** (1.3 nM agonism in HL-60 promyelocytic leukemia cells). Subsequent modifications of this scaffold involved the introduction of a naphthalene moiety into the hydrophobic portion of the molecule, which provided RARA-selective inhibitor **14** (transactivation assay EC_50_ RARA = 2.8 nM, RARB = 250 nM, RARG = 2200 nM [57]. Based on these observations, the related quinolone derivatives **15**–**17** (Table 4) were designed to increase potency and selectivity [58]. Analog **15** was found to be a potent and selective antagonist (494-fold selective for RARA over RARB and 700-fold selective over RARG). Analog **15** is the most potent and selective RARA antagonist discovered to date.

## Future directions

The orally bioavailable pan-antagonist **1** (**BMS-189453**) has provided proof of principle that inhibition of RAR leads to infertility in male mice and that this is a reversible process and well tolerated. Genetic studies underscore the critical role of RARA in particular in spermatogenesis and hence male fertility; however, an effective RARA-specific antagonist that is orally bioavailable has yet to be identified. Therefore, future studies should focus on discovering and evaluating orally active RARA-selective antagonists. RARA-specific antagonists are expected to possess marked effects on spermatogenesis and not display any possible side effects that might be due to the inhibition of the RARB or RARG. The highly lipophilic ligand-binding sites of RARs favor lipophilic ligands for binding. However, higher lipophilicity can be associated with high metabolic liability, poor oral bioavailability, high plasma protein binding, in vivo toxicity, and target promiscuity. RARA-selective antagonists with lower lipophilicity would most likely be better suited for clinical applications. Given the preference for oral administration of male contraceptives, antagonists should be designed to maximize oral bioavailability [59]. This could be accomplished by judicious application of structure filters like Lipinski’s rule of five and Veber’s rule [60, 61]. Since the structural requirements for antagonism and selectivity have been established, additional agonists could be structurally modified to become antagonists that are selective for RARA in a manner similar to the discussion above [46–48]. In addition, RARA selectivity could be sought by introducing polar linkers (such as hydrogen bond donors) to pan-antagonists to facilitate interaction with Ser232 of RARA.

## Notes

**Grant Support:** This work was supported by National Institute of Child Health and Human Development Grants U01-HD060479 and U54-HD093540/P50-HD093540, project 1 (to D.J.W. and S.S.W.C.) and HHSN275201300017C (to G.I.G) and U54-HD093540/P50-HD093540 (to G.I.G.).

## References

[1] Bearak J, Popinchalk A, Alkema L, Sedgh G Global, regional, and subregional trends in unintended pregnancy and its outcomes from 1990 to 2014: Estimates from a Bayesian hierarchical model. Lancet Glob Health 2018; 6:e380–e389. 29519649 10.1016/S2214-109X(18)30029-9 PMC6055480

[2] Long JE, Lee MS, Blithe DL Male contraceptive development: Update on novel hormonal and nonhormonal methods. Clin Chem 2019; 65:153–160. 30602479 10.1373/clinchem.2018.295089

[3] Blithe DL Pipeline for contraceptive development. Fertil Steril 2016; 106:1295–1302. 27523300 10.1016/j.fertnstert.2016.07.1115 PMC5159203

[4] Amory JK, Page ST, Bremner WJ Drug insight: Recent advances in male hormonal contraception. Nat Clin Pract Endocrinol Metab 2006; 2:32–41. 16932251 10.1038/ncpendmet0069

[5] Page ST, Amory JK, Bremner WJ Advances in male contraception. Endocr Rev 2008; 29:465–493. 18436704 10.1210/er.2007-0041 PMC2528850

[6] Liu PY, Swerdloff RS, Anawalt BD, Anderson RA, Bremner WJ, Elliesen J, Gu YQ, Kersemaekers WM, McLachlan RI, Meriggiola MC, Nieschlag E, Sitruk-Ware R et al. Determinants of the rate and extent of spermatogenic suppression during hormonal male contraception: An integrated analysis. J Clin Endocrinol Metab 2008; 93:1774–1783. 18303073 10.1210/jc.2007-2768 PMC5393365

[7] Xu L, Freeman G, Cowling BJ, Schooling CM Testosterone therapy and cardiovascular events among men: A systematic review and meta-analysis of placebo-controlled randomized trials. BMC Med 2013; 11:108. 23597181 10.1186/1741-7015-11-108 PMC3648456

[8] Westaby D, Ogle SJ, Paradinas FJ, Randell JB, Murray-Lyon IM Liver damage from long-term methyltestosterone. Lancet 1977; 310:261–263. 69876

[9] Coviello AD, Kaplan B, Lakshman KM, Chen T, Singh AB, Bhasin S Effects of graded doses of testosterone on erythropoiesis in healthy young and older men. J Clin Endocrinol 2008; 93:914–919. 10.1210/jc.2007-1692 PMC2266950 18160461

[10] World Health Organisation Task Force on Methods for the Regulation of Male Fertility Contraceptive efficacy of testosterone-induced azoospermia in normal men. Lancet 1990; 336:955–959. 1977002

[11] Blithe D Male contraception: What is on the horizon? Contraception 2008; 78:S23–S27. 18847595 10.1016/j.contraception.2008.03.018

[12] Hogarth CA, Griswold MD The key role of vitamin A in spermatogenesis. J Clin Invest 2010; 120:956–962. 20364093 10.1172/JCI41303 PMC2846058

[13] Griswold MD Spermatogenesis: The commitment to meiosis. Physiol Rev 2016; 96:1–17. 26537427 10.1152/physrev.00013.2015 PMC4698398

[14] Hogarth CA, Amory JK, Griswold MD Inhibiting vitamin A metabolism as an approach to male contraception. Trends Endocrinol Metab 2011; 22:136–144. 21277790 10.1016/j.tem.2011.01.001 PMC3070762

[15] Busada JT, Chappell VA, Niedenberger BA, Kaye EP, Keiper BD, Hogarth CA, Geyer CB Retinoic acid regulates kit translation during spermatogonial differentiation in the mouse. Dev Biol 2015; 397:140–149. 25446031 10.1016/j.ydbio.2014.10.020 PMC4268412

[16] Peer NR, Law SM, Murdoch B, Goulding EH, Eddy EM, Kim K Germ cell-specific retinoic acid receptor alpha functions in germ cell organization, meiotic integrity, and spermatogonia. Endocrinology 2018; 159:3403–3420. 30099545 10.1210/en.2018-00533 PMC6112597

[17] Endo T, Mikedis MM, Nicholls PK, Page DC, de Rooij DG Retinoic acid and germ cell development in the ovary and testis. Biomolecules 2019; 9:775. 10.3390/biom9120775 PMC6995559 31771306

[18] Chung S, Sung W, Wang X, Wolgemuth D Retinoic acid receptor alpha is required for synchronization of spermatogenic cycles and its absence results in progressive breakdown of the spermatogenic process. Dev Dyn 2004; 230:754–766. 15254909 10.1002/dvdy.20083 PMC3785309

[19] Chung SSW, Wang X, Wolgemuth DJ Male sterility in mice lacking retinoic acid receptor alpha involves specific abnormalities in spermiogenesis. Differentiation 2005; 73:188–198. 15901285 10.1111/j.1432-0436.2005.00018.x PMC3785313

[20] Chung SS, Wolgemuth DJ Role of retinoid signaling in the regulation of spermatogenesis. Cytogenet Genome Res 2004; 105:189–202. 15237207 10.1159/000078189 PMC3803148

[21] Wolgemuth DJ, Chung SS Retinoid signaling during spermatogenesis as revealed by genetic and metabolic manipulations of retinoic acid receptor alpha. Soc Reprod Fertil Suppl 2007; 63:11–23. 17566257 PMC3796155

[22] Chung SSW, Wang X, Wolgemuth DJ Expression of retinoic acid receptor alpha in the germline is essential for proper cellular association and spermiogenesis during spermatogenesis. Development 2009; 136:2091–2100. 19465599 10.1242/dev.020040 PMC2685727

[23] Cunningham TJ, Duester G Mechanisms of retinoic acid signalling and its roles in organ and limb development. Nat Rev Mol Cell Biol 2015; 16:110–123. 25560970 10.1038/nrm3932 PMC4636111

[24] Howell JM, Thompson JN, Pitt GAJ Histology of the lesions produced in the reproductive tract of animals fed a diet deficient in vitamin A alcohol but containing vitamin A acid, I. The male rat. J Reprod Fertil 1963; 5:159–167. 13955162 10.1530/jrf.0.0050159

[25] Wolbach SB, Howe PR Tissue changes following deprivation of fat-soluble A vitamin. J Exp Med 1925; 42:753–777. 19869087 10.1084/jem.42.6.753 PMC2131078

[26] Eskild W, Hansson V Vitamin A functions in the reproductive organs In: Blomhoff R (ed.), Vitamin A in Health and Disease. New York: Dekker; 1994: 531–559.

[27] Heller CG, Moore DJ, Paulsen CA Suppression of spermatogenesis and chronic toxicity in men by a new series of bis(dichloroacetyl) diamines. Toxicol Appl Pharmacol 1961; 3:1–11. 13713106 10.1016/0041-008x(61)90002-3

[28] Amory JK Development of novel male contraceptives. Clin Transl Sci 2020; 13:228–237. 31618525 10.1111/cts.12708 PMC7070810

[29] Amory JK, Muller CH, Shimshoni JA, Isoherranen N, Paik J, Moreb JS, Amory DW Sr, Evanoff R, Goldstein AS, Griswold MD Suppression of spermatogenesis by bisdichloroacetyldiamines is mediated by inhibition of testicular retinoic acid biosynthesis. J Androl 2011; 32:111–119. 20705791 10.2164/jandrol.110.010751 PMC3370679

[30] Paik J, Haenisch M, Muller CH, Goldstein AS, Arnold S, Isoherranen N, Brabb T, Treuting PM, Amory JK Inhibition of retinoic acid biosynthesis by the bisdichloroacetyldiamine WIN 18,446 markedly suppresses spermatogenesis and alters retinoid metabolism in mice. J Biol Chem 2014; 289:15104–15117. 24711451 10.1074/jbc.M113.540211 PMC4031560

[31] Chen Y, Zhu J-Y, Hong KH, Mikles DC, Georg GI, Goldstein AS, Amory JK, Schönbrunn E Structural basis of ALDH1A2 inhibition by irreversible and reversible small molecule inhibitors. ACS Chem Biol 2018; 13:582–590. 29240402 10.1021/acschembio.7b00685 PMC6089219

[32] Lufkin T, Lohnes D, Mark M, Dierich A, Gorry P, Gaub MP, LeMeur M, Chambon P High postnatal lethality and testis degeneration in retinoic acid receptor alpha mutant mice. Proc Natl Acad Sci U S A 1993; 90:7225–7229. 8394014 10.1073/pnas.90.15.7225 PMC47109

[33] Endo T, Freinkman E, de Rooij DG, Page DC Periodic production of retinoic acid by meiotic and somatic cells coordinates four transitions in mouse spermatogenesis. Proc Natl Acad Sci 2017; 114:E10132. 29109271 10.1073/pnas.1710837114 PMC5703301

[34] Schulze GE, Clay RJ, Mezza LE, Bregman CL, Buroker RA, Frantz JD BMS-189453, a novel retinoid receptor antagonist, is a potent testicular toxin. Toxicol Sci 2001; 59:297–308. 11158723 10.1093/toxsci/59.2.297

[35] Chung SS, Wang X, Roberts SS, Griffey SM, Reczek PR, Wolgemuth DJ Oral administration of a retinoic acid receptor antagonist reversibly inhibits spermatogenesis in mice. Endocrinology 2011; 152:2492–2502. 21505053 10.1210/en.2010-0941 PMC3100616

[36] Chung SS, Wang X, Wolgemuth DJ Prolonged oral administration of a pan-retinoic acid receptor antagonist inhibits spermatogenesis in mice with a rapid recovery and changes in the expression of influx and efflux transporters. Endocrinology 2016; 157:1601–1612. 26812157 10.1210/en.2015-1675 PMC4816726

[37] Teng M, Duong TT, Johnson AT, Klein ES, Wang L, Khalifa B, Chandraratna RA Identification of highly potent retinoic acid receptor alpha-selective antagonists. J Med Chem 1997; 40:2445–2451. 9258350 10.1021/jm9703911

[38] Chung SS, Cuellar RA, Wang X, Reczek PR, Georg GI, Wolgemuth DJ Pharmacological activity of retinoic acid receptor alpha-selective antagonists in vitro and in vivo. ACS Med Chem Lett 2013; 4:446–450. 24040487 10.1021/ml300365k PMC3770188

[39] Loeliger P, Bollag W, Mayer H Arotinoids, a new class of highly-active retinoids. Eur J Med Chem 1980; 15:9–15.

[40] Germain P, Iyer J, Zechel C, Gronemeyer H Co-regulator recruitment and the mechanism of retinoic acid receptor synergy. Nature 2002; 415:187–192. 11805839 10.1038/415187a

[41] Maire A le, Teyssier C, Erb C, Grimaldi M, Alvarez S, de Lera AR, Balaguer P, Gronemeyer H, Royer CA, Germain P, Bourguet W A unique secondary-structure switch controls constitutive gene repression by retinoic acid receptor. Nat Struct Mol Biol 2010; 17:801–U843. 20543827 10.1038/nsmb.1855

[42] Klein ES, Wang JW, Khalifa B, Gavigan SA, Chandraratna RAS Recruitment of nuclear receptor corepressor and coactivator to the retinoic acid receptor by retinoid ligands—influence of DNA-heterodimer interactions. J Biol Chem 2000; 275:19401–19408. 10777502 10.1074/jbc.M002472200

[43] Bourguet W, Germain P, Gronemeyer H Nuclear receptor ligand-binding domains three-dimensional structures, molecular interactions and pharmacological implications. Trends Pharmacol Sci 2000; 21:381–388. 11050318 10.1016/s0165-6147(00)01548-0

[44] Bourguet W, Ruff M, Chambon P, Gronemeyer H, Moras D Crystal-structure of the ligand-binding domain of the human nuclear receptor RXR-alpha. Nature 1995; 375:377–382. 7760929 10.1038/375377a0

[45] Bourguet W, Vivat V, Wurtz J-M, Chambon P, Gronemeyer H, Moras D Crystal structure of a heterodimeric complex of RAR and RXR ligand-binding domains. Mol Cell 2000; 6:289–298. 10.1016/s1097-2765(00)80424-4 10882070

[46] Kaneko S, Kagechika H, Kawachi E, Hashimoto Y, Shudo K Retinoid antagonists. Med Chem Res 1991; 1:220–225.

[47] Yoshimura H, Nagai M, Hibi S, Kikuchi K, Abe S, Hida T, Higashi S, Hishinuma I, Yamanaka T A novel type of retinoic acid receptor antagonist—synthesis and structure-activity-relationships of heterocyclic ring-containing benzoic-acid derivatives. J Med Chem 1995; 38:3163–3173. 7636879 10.1021/jm00016a020

[48] Hughes NE, Bleisch TJ, Jones SA, Richardson TI, Doti RA, Wang Y, Stout SL, Durst GL, Chambers MG, Oskins JL, Lin C, Adams LA et al. Identification of potent and selective retinoic acid receptor gamma (RARγ) antagonists for the treatment of osteoarthritis pain using structure based drug design. Bioorg Med Chem Lett 2016; 26:3274–3277. 27261179 10.1016/j.bmcl.2016.05.056

[49] Germain P, Kammerer S, Perez E, Peluso-Iltis C, Tortolani D, Zusi FC, Starrett J, Lapointe P, Daris JP, Marinier A, de Lera AR, Rochel N et al. Rational design of RAR-selective ligands revealed by RAR beta crystal structure. EMBO Rep 2004; 5:877–882. 15319780 10.1038/sj.embor.7400235 PMC1299136

[50] de Lera AR, Bourguet W, Altucci L, Gronemeyer H Design of selective nuclear receptor modulators: RAR and RXR as a case study. Nat Rev Drug Discov 2007; 6:811–820. 17906643 10.1038/nrd2398

[51] Gehin M, Vivat V, Wurtz JM, Losson R, Chambon P, Moras D, Gronemeyer H Structural basis for engineering of retinoic acid receptor isotype-selective agonists and antagonists. Chem Biol 1999; 6:519–529. 10421757 10.1016/S1074-5521(99)80084-2

[52] Ostrowski J, Roalsvig T, Hammer L, Marinier A, Starrett JE Jr, Yu KL, Reczek PR Serine 232 and methionine 272 define the ligand binding pocket in retinoic acid receptor subtypes. J Biol Chem 1998; 273:3490–3495. 9452473 10.1074/jbc.273.6.3490

[53] Apfel C, Bauer F, Crettaz M, Forni L, Kamber M, Kaufmann F, LeMotte P, Pirson W, Klaus M A retinoic acid receptor alpha antagonist selectively counteracts retinoic acid effects. Proc Natl Acad Sci U S A 1992; 89:7129–7133. 1323127 10.1073/pnas.89.15.7129 PMC49659

[54] Johnson AT, Klein ES, Gillett SJ, Wang LM, Song TK, Pino ME, Chandraratna RAS Synthesis and characterization of a highly potent and effective antagonist of retinoic acid receptors. J Med Chem 1995; 38:4764–4767. 7490725 10.1021/jm00024a003

[55] Johnson AT, Wang LM, Gillett SJ, Chandraratna RAS High affinity retinoic acid receptor antagonists: Analogs of AGN 193109. Bioorg Med Chem Lett 1999; 9:573–576. 10098666 10.1016/s0960-894x(99)00047-5

[56] Johnson AT, Wang LM, Standeven AM, Escobar M, Chandraratna RAS Synthesis and biological activity of high-affinity retinoic acid receptor antagonists. Biorg Med Chem 1999; 7:1321–1338. 10.1016/s0968-0896(99)00055-3 10465407

[57] Hibi S, Tagami K, Kikuchi K, Yoshimura H, Tai K, Hida T, Tokuhara N, Yamauchi T, Nagai M Syntheses and evaluation of naphthalenyl- and chromenyl-pyrrolyl-benzoic acids as potent and selective retinoic acid receptor alpha agonists. Bioorg Med Chem Lett 2000; 10:623–625. 10762039 10.1016/s0960-894x(00)00067-6

[58] Kikuchi K, Tagami K, Hibi S, Yoshimura H, Tokuhara N, Tai K, Hida T, Yamauchi T, Nagai M Syntheses and evaluation of quinoline derivatives as novel retinoic acid receptor a antagonists. Bioorg Med Chem Lett 2001; 11:1215–1218. 11354380 10.1016/s0960-894x(01)00177-9

[59] Martin CW, Anderson RA, Cheng L, Ho PC, van der Spuy Z, Smith KB, Glasier AF, Everington D, Baird DT Potential impact of hormonal male contraception: Crosscultural implications for development of novel preparations. Hum Reprod 2000; 15:637–645. 10686211 10.1093/humrep/15.3.637

[60] Lipinski CA Drug-like properties and the causes of poor solubility and poor permeability. J Pharmacol Toxicol Methods 2000; 44:235–249. 11274893 10.1016/s1056-8719(00)00107-6

[61] Veber DF, Johnson SR, Cheng H-Y, Smith BR, Ward KW, Kopple KD Molecular properties that influence the oral bioavailability of drug candidates. J Med Chem 2002; 45:2615–2623. 12036371 10.1021/jm020017n

[62] Russell LD, Ettlin RA, Hikim APS, Clegg ED Histological and histopathological evaluation of the testis. Int J Androl 1993; 16:83–83.

